# In Situ Synthesis of Reduced Graphene Oxide and Gold Nanocomposites for Nanoelectronics and Biosensing

**DOI:** 10.1007/s11671-010-9806-8

**Published:** 2010-10-06

**Authors:** Xiaochen Dong, Wei Huang, Peng Chen

**Affiliations:** 1 Key Laboratory for Organic Electronics and Information Displays (KLOEID) and Institute of Advanced Materials (IAM), Nanjing University of Posts and Telecommunications (NUPT), 9 Wenyuan Road, 210046, Nanjing, China; 2 School of Chemical and Biomedical Engineering, Nanyang Technological University, 637457, Singapore

**Keywords:** Graphene oxide, Gold nanoparticles, Nanoelectronics, Biosensing

## Abstract

In this study, an in situ chemical synthesis approach has been developed to prepare graphene–Au nanocomposites from chemically reduced graphene oxide (rGO) in aqueous media. UV–Vis absorption, atomic force microscopy, scanning electron microscopy, transmission electron microscopy, and Raman spectroscopy were used to demonstrate the successful attachment of Au nanoparticles to graphene sheets. Configured as field-effect transistors (FETs), the as-synthesized single-layered rGO-Au nanocomposites exhibit higher hole mobility and conductance when compared to the rGO sheets, promising its applications in nanoelectronics. Furthermore, we demonstrate that the rGO-Au FETs are able to label-freely detect DNA hybridization with high sensitivity, indicating its potentials in nanoelectronic biosensing.

## Introduction

Graphene, a single-layer of carbon atoms densely compacted into a two-dimensional honeycomb crystal lattice, has attracted tremendous attention in recent years, because of its unique electronic, optical, thermal, and mechanical properties [1-9]. It provides a variety of novel applications such as field-effect transistors (FETs) [10,11], ultrasensitive sensors [12,13], transparent electrodes [14], and novel nanocomposites [15,16]. Graphene is particularly advantageous in biosensing due to its large detection area, high charge mobility, low noise, and biocompatibility [17].

The common fabrication methods of graphene sheets include mechanical exfoliation from graphite [18], chemical exfoliation (chemical oxidation of graphite and subsequent reduction of the exfoliated graphite oxide sheets) [19], and epitaxial growth on the surface of silicon carbide crystals or metal substrates [20,21]. Among these methods, chemical exfoliation is particularly advantageous for low-cost, large-scale, high-yield preparation of graphene sheets. However, the electronic properties of the graphene sheets obtained by this method are not good enough for nanoelectronics. In order to enhance the electrical properties of the chemically reduced GO (rGO) sheets, many approaches have been investigated, such as changing the oxidation methods [22], reduction with different reduction agents or reduction conditions [23,24], and modification with metal nanoparticles [25].

In this study, we report a simple method for in situ synthesis of rGO-Au nanocomposites. With the assistance of sodium dodecyl sulfate (SDS), the resultant rGO-Au nanocomposites can form stable dispersion in water. Au nanoparticles formed uniformly on the rGO surface. The as-prepared rGO-Au hybrids show significantly higher hole mobility than Au-free rGO sheets. Furthermore, we demonstrate that that field-effect transistors (FETs) based on rGO-Au nanocomposites were able to detect DNA hybridization with high sensitivity, indicating its promising potentials in printable nanoelectronics and bio-nanoelectronics [26].

## Experimental

### Materials

Nature graphite flakes were obtained from NGS, Germany. Also, 98% H_2_SO_4_, 30% H_2_O_2_, potassium permanganate (KMnO_4_), and other reagents were of analytical grade and used as received. Distilled water (18 MΩ cm) from a Millipore system was used in all studies. The sequences of the DNA strands used in the experiments are as follows: probe DNA: 5'-AGG-TCG-CCG-CCC-SH-3', target DNA: 3'-TCC-AGC-GGC-GGG-5', single-base mismatched DNA: 3'-TCC-AGC-GGC-GTG-5'

### Synthesis of Graphene Oxide (GO)

GO was synthesised by a modified Hummers methods [27]. Three grams of graphite was added to 12 ml H_2_SO_4_ and kept at 80°C for 4.5 h. After sonication at room temperature, the solution was filtered using 200-nm porous filter and obtained the pre-oxidized graphite powder. To exfoliate the pre-oxidized graphite powder into monolayer graphene sheets, 2 g powder and 15 g KMnO_4_ were added into 120 ml H_2_SO_4_ with stirring and an ice-water bath was adopted to ensure the temperature remain below 10°C. The mixture was stirred for 2 h under ice-water bath. About 250 ml distilled water and 20 ml H_2_O_2_ (30%) were added to dilute the solution at room temperature. The suspending solution was precipitated for 12 h and the upper supernatant was collected and centrifuged. Successively, the GO powders were washed with 10% HCl and distilled water three times. The obtained GO was dispersed in distilled water to get a stable brown solution.

### Preparation and Characterization of rGO-Au Nanocomposites

Prior to the deposition of Au nanoparticles, the GO sheets were chemically reduced assisted by microwave irradiation (MWI). In a typical experiment of chemical reduction of GO, 3 μl of hydrazine and 0.1 g SDS were added to 20 ml of GO aqueous solution (0.5 mg/ml). After rigorous shaking, the mixture was heated with microwave irradiation (900 W, 2.40 GHz) for 30 s and obtained a stable rGO sheets dispersions. The reduced GO sheets were centrifuged and washed three times with distilled water to remove the hydrate residue, and dissolved into the SDS aqueous solution again. Au nanoparticles were deposited on rGO sheets by a chemical reduction of HAuCl_4_ in aqueous solution. In a typical procedure, 0.1 ml aqueous of 0.05 M HAuCl_4_ was added into 5 ml rGO SDS aqueous dispersion in a 10-ml bottle under rigorous stirring for 1 h. After the reduction reaction, the stable graphene–Au dispersion was obtained. The UV–Vis absorption curves of the resulting dispersions were measured using a UV–Vis spectrometer. The morphology of the rGO-Au nanocomposites was observed by transmission electron microscopy (TEM), tapping mode atomic force microscope (AFM), and scanning electron microscopy (SEM). Raman spectra were obtained with a WITec CRM200 confocal Raman microscopy system (laser wavelength 488 nm and laser spot size about 0.5 mm); the Si peak at 520 cm^-1^ was used as a reference for wavenumber calibration.

### Fabrication and Characterization of rGO-Au FETs

The rGO or rGO-Au sheets were deposited by dip-coating method onto Si substrate with 300 nm thermal oxide on top first. To fabricate bottom-gated graphene FETs, selected monolayer rGO or rGO-Au sheet was located under a microscope, and then covered by a copper grid hard mask. The gold source and drain electrodes were subsequently deposited onto the sheets by thermal evaporation. The channel length between source and drain electrodes is 20 μm, and the thickness of gold electrode is about 50 nm. The electrical measurements were performed in ambient condition using a Keithley semiconductor parameter analyzer (model 4200-SCS). The effective carrier mobility was calculated using μ = (*L*/*WC*_ox_*V*_d_)(Δ*I*_d_/Δ*V*_g_) [28], where *L* and *W* are the channel length and width, *C*_ox_ the gate oxide capacitance, *V*_d_ the source–drain voltage, *I*_d_ the source–drain current, and *V*_g_ the gate voltage. The linear regime of the transfer characteristics was used to obtain the slope Δ*I*_d_/Δ*V*_g_.

### Label-Free Electrical Detection of DNA Hybridization

It has been reported that the single-strand DNA with –SH end group can react with Au nanoparticles and can be used to realize DNA sensing [29]. To immobilize probe DNAs, the rGO-Au devices were immersed in 1 μM probe DNA solution for a period of 16 h. The samples were washed with PBS buffer to remove the weakly bound DNA, dried and characterized its electrical properties. Then, ten microliter target or mismatch DNA solution (200 nM) was pipetted onto the probe DNA decorated devices to hybridize 1 h, washed, dried, and characterized its electrical properties.

## Results and Discussion

**Figure 1:**
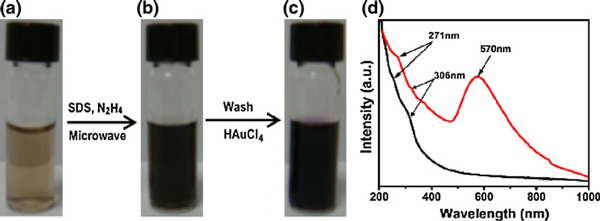
**Photographs of *(a)* GO dispersion in water, *(b)* rGO dispersion in SDS solution, c rGO-Au nanocomposites in SDS solution**. **d** UV–Vis absorptions of rGO (*black line*) and rGO-Au nanocomposites (*red line*) in SDS solution.

Figure 1 shows the photographs of GO aqueous dispersion (0.5 mg/ml), rGO, and rGO-Au aqueous dispersion in presence of SDS. GO sheets form stable brown dispersion in water (Figure 1a), because the hydroxyl, epoxy, and carboxyl groups on the GO sheets render them hydrophilic [30]. However, the reduced GO sheets tend to form irreversible agglomerates and even restack to form graphite in water because of the π–π stacking interactions between rGO sheets. In order to obtain single- or few-layer graphene sheets, various methods have been attempted using polymers [31] or aromatic molecules [26] to prevent rGO aggregation. Here, we used SDS surfactant to obtain stable rGO aqueous dispersion (Figure 1b). However, precipitation occurs after a few days. After the modification of rGO with Au nanoparticles, the resulting rGO-Au is very stable (Figure 1c) (no precipitation at all in months). It is because the Au nanoparticles on the rGO sheets act as spacers to prevent the stacking interaction [32]. It is useful for the development of printable graphene nanoelectronics.

As shown in Figure 1d, the UV–Vis spectrum of the rGO dispersion in SDS solution (black line) possesses one absorption peak at ~271 nm, which is similar to rGO in water [33]. Also, there is a weak absorption peak at ~306 nm due to incomplete reduction of GO. In contrast, the adsorption spectrum of rGO-Au nanocomposite dispersion (red line) peaks at ~570 nm, indicating that the Au nanoparticles firmly attach to the surface of the rGO sheets. The reaction mechanism for Au nanoparticles formed (reduced) on the rGO sheets may be attributable to the difference of chemical potential between rGO and gold. This reaction mechanism has been proposed to explain the decoration of carbon nanotubes with metal nanoparticles [34].

**Figure 2:**
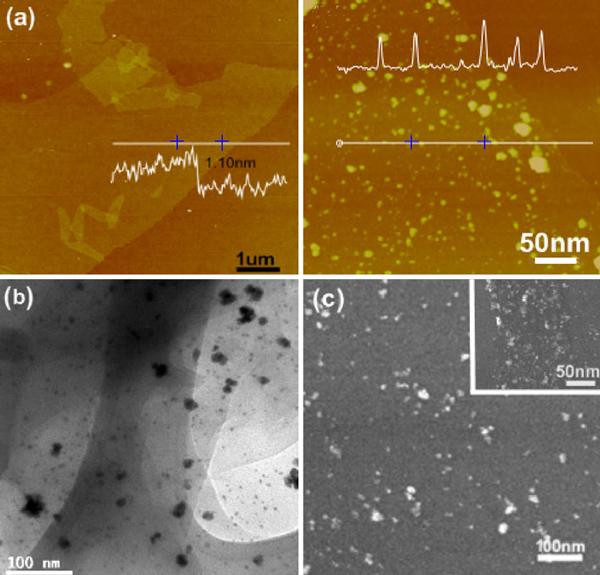
***a* AFM images of rGO sheets (*left*) and rGO sheets decorated with Au nanoparticles (*right*)**. The *insets* show the height profiles at the indicated places. ***b*** TEM image of rGO-Au sheet. ***c*** SEM image of rGO-Au sheet. The *inset* shows the view at the edge of rGO-Au sheet.

Single-layer rGO or rGO-Au sheets can be readily obtained using dip-coating of their dispersion. As revealed by AFM (Figure 2a), rGO sheet is smooth, with a thickness of ~1.10 nm which is somewhat larger than that of the mechanically exfoliated graphene monolayer (0.35 nm) due to the presence of epoxy groups on both sides of rGO sheet [35]. In contrast, numerous nanoparticles are observed on the rGO-Au sheet. As seen from Figure 2a, the Au nanoparticles (~10 nm in size) distribute uniformly on rGO sheet except a few large aggregates. TEM (Figure 2b) and SEM (Figure 2c) were also used to characterize the surface morphology of the rGO-Au sheet, where Au nanoparticles can be clearly identified. Interestingly, the density of Au nanoparticles is relatively higher at the edge of rGO sheet (Figure 2c) likely because of the higher surface energy (chemical potential) and more functional groups at the edge of rGO sheet as suggested previously [36].

**Figure 3:**
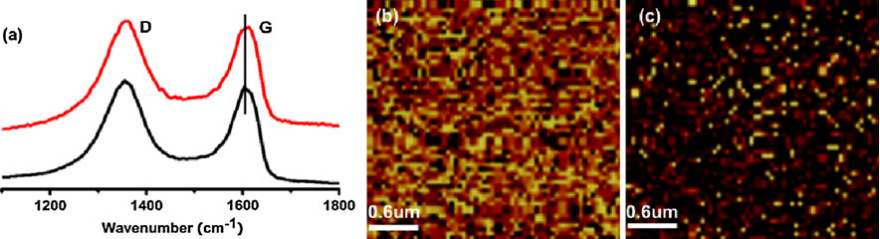
***a* Raman spectra of rGO sheet (*black line*) and rGO-Au sheet (*red line*)**. ***b*** Raman map of rGO-Au nanocomposite constructed by integrating 1,500–1,700 cm^-1^ region (G band) of graphene Raman spectrum. ***c*** Raman map of the same area constructed by integrating from 1,100 to 1,200 cm^-1^ in which the Au nanoparticles are indicated by the *bright dots*.

Figure 3a shows the Raman spectra of rGO and rGO-Au nanocomposite. The Raman spectra both display two prominent peaks at ~1,350 and ~1,600 cm^-1^, which correspond to *D* and *G* bands of graphene, respectively [37]. The *G* band corresponds to the *E*_2g_ mode and *D* band is attributed by in-plane *A*_1g_ zone-edge mode. When compared to the spectrum of rGO, the Raman *G* band slightly shifts from 1,602 to 1,606 cm^-1^ due to the *p*-doping effects imposed by the Au nanoparticles [18]. Furthermore, the 2D Raman map of rGO-Au constructed by integrating *G* band (Figure 3b) is similar to that of mechanically exfoliated single-layer graphene, indicating that our rGO-Au sheet is uniformly single-layered without significant defects. On the other hand, the 2D Raman map on the same area constructed by integrating 1,100 ~ 1,200 cm^-1^ region of Raman spectrum (Figure 3c) clearly indicates the location of Au nanoparticles (bright spots) because Au enhances Raman signal in this wavelength range [38]. Consistent with AFM, TEM and SEM imaging, these results show that Au nanoparticles are tightly attached to the surface of rGO sheets and uniformly distributed.

**Figure 4:**
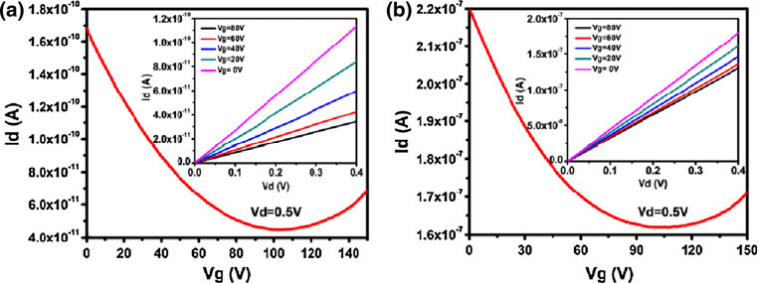
**Typical *I*_d_–*V*_g _*curve* of single-layer rGO-based FET (a) or rGO-Au-based FET (b)**. The *insets* show the *I*_d_–*V*_d _*curves* at different *V*_g_.

To investigate the effects of Au nanoparticles on the electrical properties of rGO sheets, the FETs with single-layer rGO or rGO-Au as the conducting channel were fabricated on SiO_2_/Si substrates. The linear *I*_d_–*V*_d_ curves of the devices indicate a good ohmic contact between the Au electrodes and the conducting channel (Figure 4). Both rGO FETs and rGO-Au FETs manifest typical *V*_g_-dependent ambipolar field-effect characteristics, similar to pristine graphene [1], except that the Dirac (minimum current) point significantly shift to a more positive voltage likely due to *p*-doping from oxygen or moistures on rGO. The hole mobility of the rGO and rGO-Au FETs (slope of the *I*_d_–*V*_g_ curve at *p*-region) is about 0.015 and 9.0 cm^2^/Vs, respectively. This result indicates that the hole mobility of the rGO-Au sheet is several orders higher than that of rGO sheets (8.62 ± 2.54 cm^2^/Vs vs. 0.03 ± 0.015 cm^2^/Vs, *n* = 8 devices for each case). The enhanced mobility could be explained by reduction of defect sites on rGO by decorated Au nanoparticles, because the nucleation and growth of Au nanoparticles preferentially take place at the defect sites which have higher chemical potential and more reactive functionalities [39]. Therefore, the scattering effects caused by the defects are likely relived by nanoparticles.

**Figure 5:**
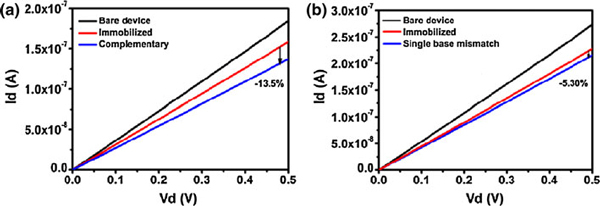
**The drain current (*I*_d_) versus drain voltage (*V*_d_) curves (at *V*_g_ = 40 V) of the bare rGO-Au device, the same device with immobilized probe DNA, and the same device after hybridization of the complementary (a) or single-base mismatched (b) DNA strands (DNA concentration is 200 nM)**.

One of the promising applications of graphene electronics is for biosensing [13]. Here, we employed our rGO-Au FETs to detect DNA hybridization. Figure 5 shows the drain current (*I*_d_) versus drain voltage (*V*_d_) curves (at *V*_g_ = 40 V) of the bare rGO-Au device, the same device with immobilized probe DNA, and the same device after hybridization of the complementary or single-base mismatched DNA strands. As shown, the drain current decrease obviously after the immobilization of thiolated probe DNA on Au nanoparticles due to *n*-doping effect of DNA molecules. After the hybridization with the complementary target DNA, the drain current decreases further about 13.5%. In a parallel experiment, the target DNA strands with single-base mismatch only caused about 5.3% decrease in drain current after hybridizing with the probe DNA. The current decease caused by the complementary DNA is significantly larger than that caused by the single-base mismatched DNA (14.07 ± 0.58% vs. 6.02 ± 0.59%, *p* < 0.001, *n* = 4 devices for each case). Therefore, our rGO-Au FET sensors are able to discriminate DNA sequence with high sensitivity. Compared to the carbon nanotube network FETs, it has the similar detection sensitivity at the same DNA concentration [40].

## Conclusions

In summary, we have demonstrated a simple approach to prepare stably dispersed rGO-Au sheets in aqueous media. Compared with the rGO sheet, the rGO-Au sheet FETs exhibit much higher hole mobility. In addition, the Au nanoparticles provide readily functionalization sites for conjugating biomolecular probes on the rGO-Au nanocomposites. We further showed that rGO-Au-based FETs are able to detect DNA hybridization with sequence specificity.
